# Editorial: Nanotechnology-based devices and systems for enhanced sensitivity and efficiency in biomedical applications

**DOI:** 10.3389/fbioe.2025.1688121

**Published:** 2025-10-02

**Authors:** Donato Conteduca, Casper Kunstmann-Olsen, Steven D. Quinn

**Affiliations:** ^1^ Center for Systems Biology, Massachusetts General Hospital, Harvard Medical School, Cambridge, MA, United States; ^2^ Mads Clausen Institute, University of Southern Denmark, Sonderborg, Denmark; ^3^ School of Physics, Engineering, and Technology, University of York, York, United Kingdom; ^4^ York Biomedical Research Institute, University of York, York, United Kingdom

**Keywords:** biomedical application (E), nanotechnology (NT), biosensing, nanofabrication, nanomaterials (ENMs)

In the last decade, we have observed an explosive increase in the use of nanotechnologies applied to important biomedical problems, driven by their ability to facilitate highly specific and selective interactions with biological targets at the nanoscale.

Technological advances in nanofabrication techniques, including scalability, integrability, and reliability of devices, are driving the biosensing evolution. In particular, they offer a remarkable contribution in overcoming typical challenges for sensing targets at the nanoscale, by controlling the interaction with the analytes with stronger and more precise modalities, as well as higher throughput with better cost-efficiency. The implementation of biosensors with biocompatible materials is fostering the translation from the lab bench to clinical practice for clinical applications and *in-vivo* studies, minimising or preventing risks for patients and facilitating the regulatory approval as medical devices (Sindhwani and Chan, 2021).

Among the wide range of biomedical and bioengineering applications that leverage nanoscale interactions with biological targets, nanotechnologies have experienced especially rapid growth in medical diagnostics and therapeutics, as well as in fundamental research in biophysics and medicine, for example, in elucidating disease onset and progression, characterising immune responses and enabling targeted drug delivery (Kirtane et al., 2021).

The integration of diverse techniques such as optical, electrical, magnetic, and microfluidic methods, with emerging materials, including nanomaterials, quantum dots, and nanostructured substrates, has been shown to significantly improve both sensing and imaging capabilities. These advances enable the investigation of a broad variety of biological targets across multiple length scales, from the microscale (i.e., cells, tissue, bacteria) to the nanoscale (e.g., proteins, nucleic acids, extracellular vesicles, and viruses) (Flynn et al., 2023; Xue et al., 2022; Altug et al., 2022; Conteduca et al., 2021; Xiong et al., 2022). Furthermore, the development of active systems capable of manipulating nanomaterial-biotarget interactions using external forces within controlled environments has led to significant advances in drug development and delivery. These systems also offer enhanced sensitivity for the precise control and investigation of increasingly smaller targets, down to the level of single molecules and nucleic acids (Ying et al., 2022). Nanotechnology-based systems have demonstrated the ability to overcome key limitations of conventional bulk systems, which often suffer from limited sensitivity, require large sample volumes and are constrained by high costs and slow processing times–factors that frequently hinder their applicability in clinical studies.

This Research Topic aimed to highlight technological innovations in the fabrication and characterisation of nanotechnology-based devices for biomedical applications, focusing on solutions that improve system integrability and reliability to facilitate the transition from the bench to clinical studies. Moreover, new theoretical and computational studies are also included to address current limitations of systems in biosensing, by taking into account effects observable in experimental activities, such as thermal effects and flow dynamics for *in-vivo* studies.

The articles published in this Research Topic investigate in greater depth several key themes and objectives of the collection. Notably, Lu et al. in their article “*Dielectrophoretic capture of Escherichia coli and boar sperms using ULSI-fabricated three-dimensional protruding TiN nano-electrode arrays*” present a nanoelectrode configuration designed to generate dielectrophoresis for the separation of live and dead sperm cells, with significant implications for fertility research. Despite the simplicity and fast response of conventional methods, they often suffer from insufficient sperm cell recovery, leading to incomplete or ambiguous outcomes. In this work, the authors implemented an array of 3D nanoelectrodes made from titanium nitride (TiN), which is a biocompatible material and suitable for semiconductor CMOS processes. These nanoelectrodes enhanced the local electric field strength by ⁓ 483%, producing a stronger dielectrophoretic effect and enabling effective cell confinement at lower applied voltages. This configuration also reduced Joule heating, further minimised by the application of an insulating layer on the metal wires, which is critical for preserving sperm motility and maintaining membrane integrity, a key factor to separate dead from live biological targets (e.g., sperms and bacteria). The authors demonstrated a maximum capture efficiency of 80% with processing rates of 2.4 mL/h and 0.2 mL/h for sperm and bacteria, respectively.

This Research Topic also features a complementary study by Liao et al. titled “*Innovative CMOS-fabricated dielectrophoretic chip: application of 3D TiN nano-electrode arrays with adjustable electrode spacing in sperm capture*”. In this work, the authors exploited the advantages offered by the 3D TiN nanoelectrode fabrication processes to improve throughput and enable faster sperm capture. The use of nanoelectrodes enhanced the local electric field by a factor of 5, maximising the dielectrophoretic force on the cells. The system incorporates a controllable 7 × 7 array of electrodes, enabling the simultaneous detection and manipulation of multiple sperm cells with a capture efficiency of 60% and a processing rate of up to 6 mL/h. Importantly, the design offers scalability, providing a clear pathway for further parallelization and integration in high-throughput diagnostic applications.

The Research Topic includes a theoretical study exploring the role of magnetic nanoparticles applied in medical diagnostics and drug delivery. Van Dieren et al. in their work “*Computational modeling of superparamagnetic nanoparticle-based (affinity) diagnostics*” employed computational modeling to investigate the flow of magnetic nanoparticles (MNPs) in blood vessels under the influence of external magnetic fields. A finite element method was used to simulate the flow dynamics of iron oxide nanoparticles (IONPs), incorporating key parameters such as viscosity and particle flow rate. The authors observed that the concentration of MNPs required for effective diagnostic performance is highly dependent on both the sensitivity of the detection scheme and the intrinsic properties of the MNPs. The authors also discussed ongoing challenges limiting the clinical translation of IONPs, particularly their limited biocompatibility. They emphasised the need for long-term *in vivo* studies to better characterise the clearance mechanisms of MNPs within the vasculature, thereby informing model refinement and minimising potential toxicity.

Finally, the Research Topic is completed with an article focusing on a surface-enhanced Raman scattering (SERS) platform for the early detection of diabetic retinopathy (DR). Zeng et al. in their work “*Nanoenzymatic SERS bifunctional detection platform based on recognition competition strategy for ultrasensitive detection of diabetic retinopathy-related biomarkers*” developed Au@PD nanorods modified with single-stranded DNA1 (ssDNA1), employed as nanoenzymatic probes. The substrate is modified with an array of Au trioctahedral (AuTOHs) modified with aptamer strands and single-stranded DNA2 (ssDNA2). In the presence of VEGF in the serum sample, one of the most critical biomarkers in the early diagnosis of DR, the target binds to the aptamer with higher affinity than the aptamer-ssDNA2 interaction. This process causes a displacement of the ssDNA2, which can hybridize with ssDNA1, with a consequent attachment of the Au@PD nanorods onto the substrate and a relevant increase of the SERS signal, proportional to the VEGF concentration.

The sensing platform showed excellent peroxidase (POD) activity, a SERS enhancement >10^9^, a limit of detection of 0.11 pg/mL, an assay time of only 14 min, and high reliability directly from complex serum samples.

This Research Topic contributes to highlighting possible solutions to consider for future directions in the field of biosensors, in terms of manufacturing reliability and improvement of sensing performance, aiming at facilitating the use of such systems in a clinical scenario for personalised medicine, with strong potential for fields such as pathology, pharmacology, and immunology. In addition to technological advances, artificial intelligence applied to data analysis is contributing remarkably to the exponential growth of the biosensing field, typically focusing on minimising the noise contribution with better sensor resolution and accuracy (Akkaş et al., 2025). Furthermore, in recent years, machine learning has also been implemented to directly optimize the design of biosensors (Rao et al., 2024). The combination of these computational methods together with high-resolution nanofabrication techniques is paving the way for the next-generation biosensors, characterized by higher reliability and more tolerance to external factors that could affect the measurements of specific biotargets, facilitating the integration of the sensing devices in cost-efficient point-of-care systems. This strategy has the potential to overcome the current challenges for the clinical translation of biosensing systems, typically associated with too high costs, the requirement of expert users and insufficient sensitivity for many pathological studies, by offering cost-efficient and accessible solutions without compromising the diagnostic accuracy.
